# Double-diffusive Hamel–Jeffrey flow of nanofluid in a convergent/divergent permeable medium under zero mass flux

**DOI:** 10.1038/s41598-023-27938-0

**Published:** 2023-01-20

**Authors:** S. Ahmad, M. Farooq

**Affiliations:** 1 Department of Mathematics and Statistics, Riphah International University, Islamabad, 44000 Pakistan; 2 Department of Pure and Applied Mathematics, The University of Haripur, Haripur, KPK Pakistan

**Keywords:** Applied mathematics, Fluid dynamics

## Abstract

In the recent era, the nanofluid's transportation due to the Jeffrey–Hemal flow phenomenon (i.e., carrying fluid through a converging/diverging channel) has significant applications in numerous engineering and science technologies. Therefore, multi-disciplinary evolution and research motivated us to present current attempt. The aim of this attempt is to present Jeffrey–Hamel mechanism of the nanofluid through non-parallel channel under thermally balance non-Darcy permeable medium impacts. The nanomaterial is represented using the Buongiorno nanofluid model. The investigation also includes zero mass flux impacts as well as variable rheological fluid properties. The influences of temperature jump are also encountered in the current analysis. The governing flow expressions under the Jeffrey–Hemal analysis are made dimensionless utilizing the similarity variables. The dimensionless equations are then solved using the analytical scheme (homotopy method) and the obtained series solutions are convergent. The influences of the involved parameters on concerned profiles are investigated through graphs. Force of drag, Nusselt and Sherwood numbers are elaborated graphically. In this analysis, intensification in Prandtl number enhances the heat transfer rate whereas decrement is seen in heat transfer rate for larger thermal slip parameter. Further, mass diffusivity parameter adversely affects the mass transfer rate. The current analysis incorporates numerous industrial and technological processes including transportation, material synthesis, microfluidics, high-power Xrays, biomedical, solid-state lighting, microelectronics, scientific measurement, medicine, molten polymers extrusion via converging dies, cold drawing operation related to polymer industry etc.

## Introduction

The initial motivation regarding enhancement in the thermal conductivity through saturation of submicronic solid type particles (i.e., nanoparticles) into liquid were described in 1993 by Masuda et al.^1^. At the first time, Choi et al.^2^ proposed a model and made use of the term" nanofluid" in order to indicate that the engineered colloids are consisted of nanoparticles which saturated into a base fluid. However, in recent, nanoparticles have become cheaper and widely obtainable which give hope their use for practical applications. For instance, possible usage of nanofluids as coolant agent can be explored for advanced nuclear systems. Contrary exploration of discovered mili-sized or micro-sized particles in the past, the size of the nanoparticles is relatively very near to the size of base fluid's molecules, and thus under little settlement in gravitation the nanoparticles show stable behavior for longer period of time. Usually materials include metals such as gold, copper, and oxides such as titania, copper oxide, alumina, silica act as the nanoparticles. Moreover, diamond as well as carbon nanotubes can also be used as nanoparticles to form the nanofluid. Likewise water and organic fluids such as ethylene glycol and ethanol are adopted as the popular base fluids. Keeping in mind the groundbreaking idea, several investigators have used different models to study the aspects of Brownian motion and thermophoresis diffusion. Mohyud-Din et al.^3^ disclosed the nanofluid motion through convergent-divergent channel by utilizing Buongiorno's model. Vinita and Poply^4^ disclosed the slip features in hydro-magnetic nanofluid caused by stretchable cylinder. Majeed et al.^5^ discussed the nanofluid motion through circular type cylinder using the Buongiorno’s model under the significance of multiple slips. Alsaedi et al.^6^ illustrated the mixed convection features in the peristalsis of magneto nanofluid under the impact of compliant wall. Laila^7^ disclosed the convergent/divergent influences on motion of nanofluid through rectangular channel with heated walls. Ayeche et al.^8^ discussed the variable magnetic characteristics in biofluid flow caused by wedge. Magnetic field describes substantial influences on the bio-magnetic flow and heat phenomena. Benaziza et al.^9^ described the irreversibility features in magneto nanofluid motion through coaxial cylindrical channel. Ohmic heating and chemical reaction incorporates to enhance the flow analysis.

Kalpana et al.^10^ reported the magnetic impacts in nanofluid flow with Brownian and thermophoretic diffusion phenomena. Habiyaremye et al.^11^ described the magnetic features in nanomaterial flow caused by convergent-divergent channel under the heat and mass transport phenomenon. Rehman et al.^12^ disclosed the Jeffrey–Hamel flow of Carreau nanoliquid under heat and mass transport analysis. Hamrelaine et al.^13^ disclosed the magnetic features in ferro-nanomaterial flow through converging/diverging rotating channel. The study witnesses that magnetic field reverses the flow behavior in both convergent/divergent channel. Biswal et al.^14^ described the motion of nanofluid caused by stretchable inclined plates. Here, the stretching/shrinking impacts play a significant role over dimensionless profiles. Results revealed that shrinking/stretching parameter helps in accelerating the velocity while decelerates the temperature. Qadeer et al.^15^ explored the converging/diverging effects in flow of nanofluid under the irreversibility analysis. The irreversibility change of system is eminent near to the channel walls.

Literature unfold that many of researches have investigated by incorporating constant physical fluid characteristics such as viscosity, thermal conductivity etc., however, variation in these physical characteristics significantly may occur in respect of temperature difference. For instance, viscosity of water falls about 240% when temperature changes from 10 to 50 °C. It means that variations in such physical quantities are needed in order to understand the flow behavior accurately. Furthermore, most analysts’ studies related to concentration are incorporating with constant mass diffusivity features. However, there are many applications in existence where mass diffusivity variation with respect to concentration is taken into consideration in certain range. Mass diffusivity variation may occur linearly or exponentially in real life application. Farooq et al.^16^ reported the variable fluid characteristics in squeezed flow through permeable media under modified heat and mass fluxes. Ferdows and Alzahrani^17^ explored the hydro-magnetic slip features in darcian flow of reactive fluid with variable properties. Gahgah et al.^18^ described the flow features of viscoelastic fluid caused by non-parallel sheets. The study concludes that higher Weissenberg number depicts reversal flow trend through converging/diverging channel. Latreche et al.^19^ disclosed the flow behavior of diverse viscoelastic liquids through flat and circular ducts. Here, PTT and FENE-P liquids become identical when pressure gradient parameters approaches to 1. Sharma and Kumawat^20^ disclosed the varying liquid properties in MHD reactive flow through a stretchable sheet considering ohmic impact. Awais et al.^21^ described the magnetic impacts in bio-convective nanomaterial motion along-with gyrotactic microorganisms and under variable thermal conductivity and mass diffusivity effects. Salahuddin et al.^22^ discussed the variable properties effect in viscoelastic liquid flow. Mottupalle et al.^23^ reported the variable properties (thermal conductivity, mass diffusivity) effect in double diffusive reactive flow caused by accelerating surface with impact of mixed convection. Waqas^24^ depicted the dual diffusive reactive flow of Maxwell liquid under the impact of variable properties i.e., thermal conductivity and mass diffusivity. Jabeen et al.^25^ explored the dual generalized fluxes in Maxwell stratified fluid under varying liquid properties. Variable thermal conductivity and mass diffusivity enhance the thermal and solutal profiles. Abbasi et al.^26^ explained the peristaltic features in magneto nanomaterial through non-uniform channel under the impact of irreversibility and varying fluid characteristics. Thermal conductivity increases the heat transfer features during peristalsis movement.

Flow phenomenon through porous medium is significant aspect in numerous areas related to reservoir engineering, for instance, environmental, petroleum and groundwater hydrology. In such areas, the design and operation of projects are considered to be successful when fluid flow through porous media has been accurately described. In many cases, Darcy's law^27^ has been utilized to describe the fluid flow through porous media. However, in some cases where the high velocity occurs, the Darcy's law faces difficulty in order to implement for the description of fluid flow. Therefore, in order to overcome the deficiency occurred in Darcy's law, Forchheimer^28^ included an extra term known as non-Darcy term which comprises density of fluid, coefficient of non-Darcy and superficial velocity. Non-Darcy behavior effectively utilizes in the industrial and engineering processes comprise grain storage, production of crude oil, mastic transport modeling, groundwater pollution, porous insulation, nuclear waste discarding and many other. In this direction, Kumar et al.^29^ discussed the non-Darcian flow analysis through convergent/divergent channel in the presence of carbon nanotubes. Alzahrani et al.^30^ described the non-Darcy analysis in hybrid radiative nanofluid flow through flat sheet. Khan et al.^31^ disclosed the Dufour and Soret features in fluid flow through permeable medium with entropy generation. Ahmad et al.^32^ analyzed the slip and non-Darcy features in hybrid nanomaterial flow under the impact of convergent/divergent channel. Muhammad et al.^33^ reported the melting effects in hybrid nanofluid inserted in non-darcy permeable medium. Ahmad et al.^34^ explored the variable properties in non-Darcina flow of second grade nanoliquid considering non-linear stratification phenomenon. Wang et al.^35^ described the entropy features in non-Darcy movement of nanofluid under the influence of thermal conductivity. Consequences show that porous medium offers resistance to the flow. Khan et al.^36^ discussed non-Darcy and Ohmic heating impacts in fluid flow under irreversibility phenomenon. The analysis shows that porosity variable decrements the flow field.

The survey of literature reveals that no article is presented for nanofluid flow through converging/diverging channel with the influence of zero mass flux and variable fluid's properties (i.e., thermal conductivity and mass diffusivity). Thus, the current work aims to analyze the viscous fluid with nanoparticles inserted in non-Darcy porous medium. The flow is subjected to converging/diverging channel. The condition of zero mass flux and temperature jump has been implemented to examine the processes of heat and mass transfer. Variable thermal conductivity and mass diffusivity are also presented. A nonlinear problem is performed analytically by utilizing homotopy technique^37,38^ and results are acquired for numerous emerging parameters. Outcomes of skin friction, Nusselt and Sherwood numbers are also explored through graphs.

## Description and formulation of the problem

### Flow analysis

**Figure 1:**
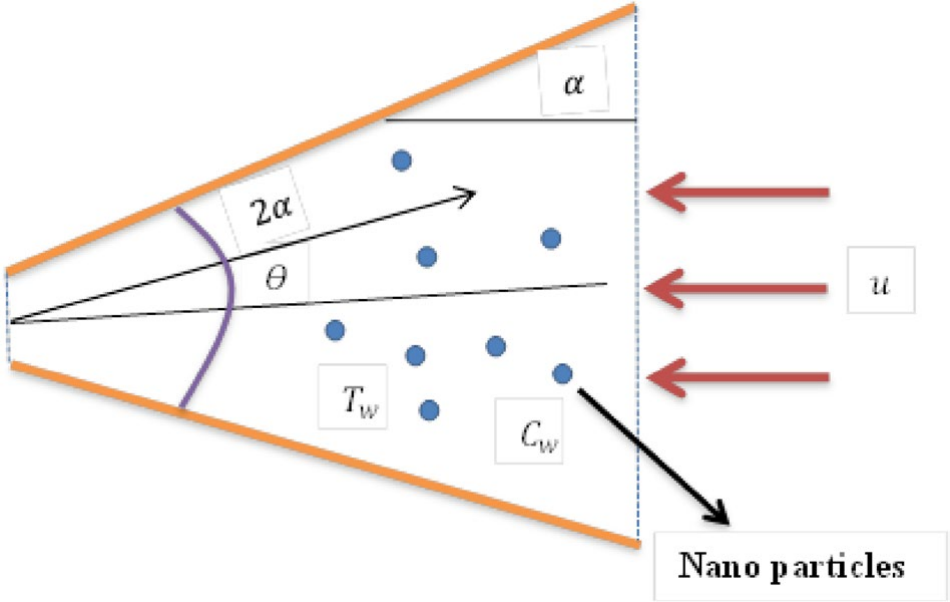
Geometrical flow description.

The model illustrates the steady flow of incompressible fluid with the inclusion of nanoparticles in a non-parallel channel subject to a non-Darcy medium and uniform pressure gradient. The channel walls are found at an inclination of $2\alpha$, as displayed in Fig. 1. In order to formulate the problem, the cylindrical polar coordinates $\left( {r,\theta ,z} \right)$ are implemented. The fluid movement is observed to be in purely radial direction, therefore, velocity of fluid takes the form as $V = u\left( {r,\theta } \right)$ i.e., the velocity field is consisted to be $r$ and $\theta$ only, whereas other components $\left( {v, w} \right)$ are considered zero. Since the flow has been taken between convergent and divergent sheets, thus, $\alpha < 0$ represents convergent channel, while the channel is divergent for $\alpha > 0$. The fluid motion is driven by pressure gradient. Slip condition is considered at wall for temperature. With these assumptions, the flow equation can be expressed as ^3^:

1 $$ \frac{{\rho_{f} }}{r}\frac{\partial }{\partial r}\left( {ru} \right) = 0, $$

2 $$ u\frac{\partial u}{{\partial r}} = \frac{ - 1}{{\rho_{f} }}\left( {\frac{\partial p}{{\partial r}}} \right) + \frac{{\mu_{f} }}{{\rho_{f} }}\left( {\frac{{\partial^{2} u}}{{\partial r^{2} }} + \frac{1}{r}\frac{\partial u}{{\partial r}} + \frac{1}{{r^{2} }}\frac{{\partial^{2} u}}{{\partial \theta^{2} }} - \frac{u}{{r^{2} }}} \right) - \frac{{\mu_{f} \phi }}{{\rho_{f} k^{*} }}u - \frac{{C_{b}^{*} \phi }}{{\sqrt {k^{*} } }}u^{2} , $$

3 $$ \frac{1}{{\rho_{f} }}\left( {\frac{\partial p}{{\partial \theta }}} \right) - \frac{2}{r}\frac{{\mu_{f} }}{{\rho_{f} }}\frac{\partial u}{{\partial \theta }} = 0, $$

where, ${\rho }_{f}$ represents fluid density, $u$ represents radial velocity, $p$ denotes pressure, ${\mu }_{f}$ represents dynamic viscosity, $\phi$ represents porous medium porosity, ${C}_{b}\left(={C}_{b}^{*}/r\right)$ denotes constant drag coefficient, and ${k}^{*}$ represents porous medium permeability.

Upon eliminating the pressure gradient from Eqs. (2) and (3), we get the following expression:

4 $$ \begin{aligned} u\frac{{\partial^{2} u}}{\partial r\partial \theta } + \frac{\partial u}{{\partial \theta }}\frac{\partial u}{{\partial r}} = & \frac{{\mu_{f} }}{{\rho_{f} }}\left( {\frac{{\partial^{3} u}}{{\partial r^{2} \partial \theta }} + \frac{1}{r}\frac{{\partial^{2} u}}{\partial r\partial \theta } + \frac{1}{{r^{2} }}\frac{{\partial^{3} u}}{{\partial \theta^{3} }} - \frac{1}{{r^{2} }}\frac{\partial u}{{\partial \theta }}} \right) - \frac{{\mu_{f} \phi }}{{\rho_{f} k^{*} }}\frac{\partial u}{{\partial \theta }} - \frac{{2C_{b}^{*} \phi }}{{\sqrt {k^{*} } }}u\frac{\partial u}{{\partial \theta }}, \\ & + \frac{2}{{r^{2} }}\frac{{\mu_{f} }}{{\rho_{f} }}\frac{\partial u}{{\partial \theta }} - \frac{2}{r}\frac{{\mu_{f} }}{{\rho_{f} }}\frac{{\partial^{2} u}}{\partial r\partial \theta }, \\ \end{aligned} $$

the associated boundary conditions are:

5 $$ \begin{gathered} u = \frac{{U_{c} }}{r}, \frac{\partial u}{{\partial \theta }} = 0,\quad at\quad \theta = 0, \hfill \\ u = U_{w} = \beta_{1} /r,\quad at\quad \theta = \pm \alpha , \hfill \\ \end{gathered} $$

since radial velocity $\left( u \right)$ is the function of $r$ and $\theta$ so that parameter of velocity can be described as:

6 $$ ru\left( {r,\theta } \right) = F\left( \theta \right), $$

Jeffrey–Hamel flow mechanism falls in dual channels where the fluid flows inside at one end called converging channel and in diverging channel the fluid removes ward at other end. Moreover, the fluid velocity is achieved its peak value at $\theta = 0$ i.e., one can have:

7 $$ U_{max} = U_{c} = F\left( 0 \right)/r, $$

subsequently, $F\left( \theta \right) \le F\left( 0 \right)$, in the range $- \alpha \le \theta \le \alpha$.

By introducing:

8 $$ \eta = \frac{\theta }{\alpha }, f\left( \eta \right) = \frac{F\left( \theta \right)}{{U_{c} }}, $$

and in view of Eq. (6), Eq. (4) gets the form:

9 $$ f^{\prime\prime\prime} + 4\alpha^{2} f^{\prime} + 2\alpha \left( {Re} \right)ff^{\prime} - \lambda \alpha \left( {Re} \right)f^{\prime} - 2\lambda_{1} \alpha \left( {Re} \right)ff^{\prime}, $$

with $$\eta \in \left[ { - 1,1} \right]$$.

Where, $\lambda \left( { = \upsilon_{f} \phi /k^{*} U_{c} } \right)$ denotes Darcy number, $\lambda_{1} \left( { = C_{b} \phi^{*} /\sqrt {k^{*} } } \right)$ denotes inertia parameter and Reynolds number represents as:

10 $$ Re = \frac{{\alpha U_{c} }}{{\upsilon_{f} }} = \left\{ {\begin{array}{*{20}c} {U_{c} > 0, \alpha > 0, {\text{Divergent channel}};} \\ { U_{c} < 0, \alpha < 0, {\text{Convergent channel}}.} \\ \end{array} } \right. $$

The associative boundary conditions are:

11 $$ f\left( \eta \right) = 1, f^{\prime}\left( \eta \right) = 0, at\; \eta = 0, $$

and at the center of channel is

12 $$ f\left( { \pm \eta } \right) = \beta , at\; \eta = 1. $$

### Heat and mass transfer analysis

This portion represents the heat and mass transportation throughout the nanofluid drift via converging and diverging channels. Here, variable fluid characteristics (thermal conductivity and mass diffusivity) and thermal jump are present in the system, and the zero mass flux effects, then energy and concentration equations can be proposed as^3^:

13 $$ \begin{aligned} u\frac{\partial T}{{\partial r}} = & \frac{{k_{\infty } \in }}{{\left( {\rho C_{p} } \right)_{f} r}}\frac{\partial T}{{\partial r}}g\left( \eta \right) + \frac{{k_{\infty } \in }}{{\left( {\rho C_{p} } \right)_{f} r^{2} \alpha }}\frac{\partial T}{{\partial \theta }}g^{\prime}\left( \eta \right) + \frac{{k_{\infty } }}{{\left( {\rho C_{p} } \right)_{f} }}\left( {1 + \in g\left( \eta \right)} \right)\left( {\frac{{\partial^{2} T}}{{\partial r^{2} }} + \frac{1}{r}\frac{\partial T}{{\partial r}} + \frac{1}{{r^{2} }}\frac{{\partial^{2} T}}{{\partial \theta^{2} }}} \right) \\ & + \tau D_{B} \left( {\frac{\partial T}{{\partial r}}\frac{\partial C}{{\partial r}} + \frac{1}{{r^{2} }}\frac{\partial T}{{\partial \theta }}\frac{\partial C}{{\partial \theta }}} \right) + \frac{{\tau D_{T} }}{{T_{1} }}\left( {\left( {\frac{\partial T}{{\partial r}}} \right)^{2} + \frac{1}{{r^{2} }}\left( {\frac{\partial T}{{\partial \theta }}} \right)^{2} } \right), \\ \end{aligned} $$

14 $$ \begin{aligned} u\frac{\partial C}{{\partial r}} = & \frac{{D_{\infty } \in_{1} }}{r}\frac{\partial C}{{\partial r}}h\left( \eta \right) + \frac{{D_{\infty } \in_{1} }}{{r^{2} \alpha }}\frac{\partial C}{{\partial \theta }}h^{\prime}\left( \eta \right) + D_{\infty } \left( {1 + \in_{1} h\left( \eta \right)} \right)\left( {\frac{{\partial^{2} C}}{{\partial r^{2} }} + \frac{1}{r}\frac{\partial C}{{\partial r}} + \frac{1}{{r^{2} }}\frac{{\partial^{2} C}}{{\partial \theta^{2} }}} \right) \\ & + \frac{{D_{T} }}{{T_{1} }}\left( {\frac{{\partial^{2} T}}{{\partial r^{2} }} + \frac{1}{r}\frac{\partial T}{{\partial r}} + \frac{1}{{r^{2} }}\frac{{\partial^{2} T}}{{\partial \theta^{2} }}} \right), \\ \end{aligned} $$

with the boundary conditions:

15 $$ \begin{gathered} \frac{\partial T}{{\partial \theta }} = 0,\quad \frac{\partial C}{{\partial \theta }} = 0,\quad at\quad \theta = 0, \hfill \\ T = T_{w} - \beta \frac{\partial T}{{\partial \theta }},\quad D_{B} \frac{\partial C}{{\partial r}} + \frac{{D_{T} }}{{T_{1} }}\frac{\partial C}{{\partial r}} = 0\quad at\quad \theta = \pm \alpha , \hfill \\ \end{gathered} $$

Here, $T$ represents temperature, ${k}_{\infty }$ represents surrounding thermal conductivity, ${\rho }_{f}$ represents fluid density, ${\left({C}_{p}\right)}_{f}$ represents heat capacity, $\alpha$ represents channel angle, ${T}_{1}$ represents ambient temperatures, ${D}_{B}$ represents Brownian diffusion coefficient, ${D}_{T}$ denotes thermophorsis diffusion coefficient, $C$ represents concentration, $\tau \left(={\left({\rho C}_{p}\right)}_{p}/{\left({\rho C}_{p}\right)}_{f}\right)$ represents ratio of thermal capacity, $\beta$ represents thermal slip coefficient, ${D}_{\infty }$ represents surrounding mass diffusivity, and $k\left(T\right)={k}_{\infty }\left(\left(1+\in g\left(\eta \right)\right)\right)$, $D\left(C\right)={D}_{\infty }\left(1+{\in }_{1}h\left(\eta \right)\right)$ represent expressions for temperature and concentration dependent thermal conductivity and mass diffusivity, respectively, where $\left(\in , {\in }_{1}\right)$ represents small parameters known as thermal conductivity and mass diffusivity parameters, respectively, and $\left(g\left(\eta \right), h\left(\eta \right)\right)$ represent dimensionless temperature and concentration, respectively.

The dimensionless transformations are prescribed as:

16 $$ \eta = \frac{\theta }{\alpha },\quad T = \frac{{T_{w} }}{{r^{2} }}g\left( \eta \right),\quad C = \frac{{C_{w} }}{{r^{2} }}h\left( \eta \right), $$

Incorporating Eq. (16), into Eqs. (13)–(15), we have:

17 $$ \begin{gathered} g^{\prime\prime} + 4\alpha^{2} g + \in gg^{\prime\prime} + \in g^{{\prime}{2}} + 2\alpha^{2} \in g^{2} + 2\alpha \left( {Re} \right)\left( {Pr} \right)fg + \left( {Pr} \right)N_{b} \left( {g^{\prime}h^{\prime} + 4\alpha^{2} gh} \right) \hfill \\ + \left( {Pr} \right)N_{t} \left( {g^{{\prime}{2}} + 4\alpha^{2} g^{2} } \right) \hfill \\ \end{gathered} $$

18 $$ h^{\prime\prime} + 4\alpha^{2} h + \in_{1} hh^{\prime\prime} + \in_{1} h^{{\prime}{2}} + 2\alpha^{2} \in_{1} h^{2} + 2\alpha \left( {Re} \right)\left( {Le} \right)\left( {Pr} \right)fh + \left( {\frac{{N_{t} }}{{N_{b} }}} \right)\left( {g^{\prime\prime} + 4\alpha^{2} g} \right), $$

The associative boundary conditions are:

19 $$ \begin{gathered} g^{\prime}\left( \eta \right) = 0, h^{\prime}\left( \eta \right) = 0,\;at\; \eta = 0, \hfill \\ g\left( \eta \right) = 1 - \gamma g^{\prime}\left( \eta \right), N_{b} h\left( \eta \right) + N_{t} g\left( \eta \right) = 0,\;at\; \eta = 1, \hfill \\ \end{gathered} $$

Here, ${N}_{b}\left(=\tau {D}_{B}{C}_{w}/{r}^{2}{\upsilon }_{f}\right)$ denotes Brownian diffusion parameter, $Pr\left(={\mu }_{f}{C}_{p}/{k}_{\infty }\right)$ denotes Prandtl number, $Le\left(={\alpha }^{*}/{D}_{B}\right)$ where ${\alpha }^{*}$ represents thermal diffusivity, and ${N}_{t}\left(=\tau {D}_{T}{T}_{w}/{T}_{1}{r}^{2}{\upsilon }_{f}\right)$ represents thermophoretic parameter.

### Engineering Parameters

The dimensionless parameters such as coefficient of skin friction, Nusselt and Sherwood numbers can be defined as:

20 $$ C_{f} = \frac{{\mu_{f} r}}{{\rho_{f} U_{c}^{2} }}\left. {\left( {\frac{\partial u}{{\partial \theta }}} \right)} \right|_{\theta = \pm \alpha } ,\quad Nu = - \frac{{r^{2} }}{{T_{w} }}\left. {\left( {\frac{\partial T}{{\partial \theta }}} \right)} \right|_{\theta = \pm \alpha } ,\quad Sh = - \frac{{r^{2} }}{{C_{w} }}\left. {\left( {\frac{\partial C}{{\partial \theta }}} \right)} \right|_{\theta = \pm \alpha } , $$

By considering Eqs. (6), (8) and (16), Eq. (20) could be expressed as:

21 $$ \left( {Re} \right)C_{f} = f^{\prime}\left( { \pm 1} \right),\quad \alpha Nu = - g^{\prime}\left( { \pm 1} \right),\quad \alpha Sh = - h^{\prime}\left( { \pm 1} \right), $$

## Analytical solution via homotopic technique

The adopted homotopy technique is deployed to ensure the series solutions (analytical) of transformed problem defined by Eqs. (9), (17), (18) under conditions at boundary (11), (12) and (19). In this regard, the base functions $\left\{{\eta }^{k}exp\left(-c\eta \right)/k\ge 0,c\ge 0\right\}$ have been chosen. The deployed technique highly confides upon initial approximations and linear operators. Hence, we choose:

*Initial guesses*

22 $$ f_{o} \left( \eta \right) = 1 + \left( {\beta - 1} \right)\eta^{2} ,\quad g_{o} \left( \eta \right) = 1,\quad h_{o} \left( \eta \right) = - \frac{{N_{t} }}{{N_{b} }}. $$

*Linear operators*

23 $$ {\mathcal{L}}_{f} = f^{\prime\prime\prime},\quad {\mathcal{L}}_{g} = g^{\prime\prime}, \quad {\mathcal{L}}_{h} = h^{\prime\prime}. $$

The expanded form of ${\mathcal{L}}_{f} , {\mathcal{L}}_{g}$ and ${\mathcal{L}}_{h}$ are:

24 $$ {\mathcal{L}}_{f} \left( {C_{1} + C_{2} \eta + C_{3} \eta^{2} } \right) = 0,\quad {\mathcal{L}}_{g} \left( {C_{4} + C_{5} \eta } \right) = 0,\quad {\mathcal{L}}_{h} \left( {C_{6} + C_{7} \eta } \right) = 0. $$

By using Taylor’s expansion:

25 $$ \overset{\lower0.5em\hbox{$\smash{\scriptscriptstyle\smile}$}}{f} \left( {\eta ;q} \right) = f_{0} \left( \eta \right) + \mathop \sum \limits_{m = 1}^{\infty } f_{m} \left( \eta \right)q^{m} , $$

26 $$ \overset{\lower0.5em\hbox{$\smash{\scriptscriptstyle\smile}$}}{g} \left( {\eta ;q} \right) = g_{0} \left( \eta \right) + \mathop \sum \limits_{m = 1}^{\infty } g_{m} \left( \eta \right)q^{m} , $$

27 $$ \overset{\lower0.5em\hbox{$\smash{\scriptscriptstyle\smile}$}}{h} \left( {\eta ;q} \right) = h_{0} \left( \eta \right) + \mathop \sum \limits_{m = 1}^{\infty } h_{m} \left( \eta \right)q^{m} , $$

now

28 $$ \left. {f_{m} \left( \eta \right) = \frac{1}{m!}\frac{{\partial^{m} \overset{\lower0.5em\hbox{$\smash{\scriptscriptstyle\smile}$}}{f} \left( {\eta ;q} \right)}}{{\partial q^{m} }}} \right|_{q = 0} ,\quad \left. {g_{m} \left( \eta \right) = \frac{1}{m!}\frac{{\partial^{m} \overset{\lower0.5em\hbox{$\smash{\scriptscriptstyle\smile}$}}{g} \left( {\eta ;q} \right)}}{{\partial q^{m} }}} \right|_{q = 0} , \quad \left. {h_{m} \left( \eta \right) = \frac{1}{m!}\frac{{\partial^{m} \overset{\lower0.5em\hbox{$\smash{\scriptscriptstyle\smile}$}}{h} \left( {\eta ;q} \right)}}{{\partial q^{m} }}} \right|_{q = 0} . $$

The equations can also be expressed as:

29 $$ {\mathcal{L}}_{f} \left[ {f_{m} \left( \eta \right) - \chi_{m} f_{m - 1} \left( \eta \right)} \right] = \hbar_{f} R_{m}^{f} \left( \eta \right), $$

30 $$ {\mathcal{L}}_{g} \left[ {g_{m} \left( \eta \right) - \chi_{m} g_{m - 1} \left( \eta \right)} \right] = \hbar_{g} R_{m}^{g} \left( \eta \right). $$

31 $$ {\mathcal{L}}_{h} \left[ {h_{m} \left( \eta \right) - \chi_{m} h_{m - 1} \left( \eta \right)} \right] = \hbar_{h} R_{m}^{h} \left( \eta \right). $$

### Convergence discussion

**Figure 2:**
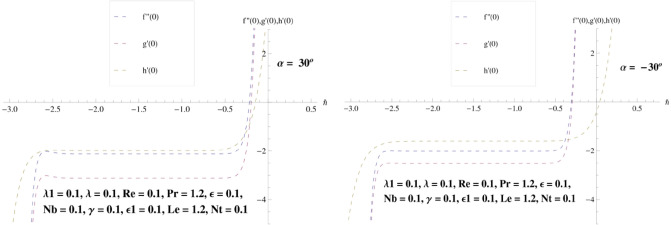
Convergence for $f\left( \eta \right),g\left( \eta \right)$ and $h\left( \eta \right)$ in case of convergent/divergent channel.

Liao [45] highlighted that the approximations rate, and convergent solutions via HAM have revealed dependence upon auxiliary parameters $\hbar_{f} , \hbar_{g}$ and $\hbar_{h}$. Hence, h-curves have been drawn with $f^{\prime\prime}\left( 0 \right)$, $g^{\prime}\left( 0 \right)$ and $h^{\prime}\left( 0 \right)$ in order to detect the suitable values of such parameters, which shows in Fig. 2. The adequate ranges for ${\hslash }_{f}, {\hslash }_{g}$ and ${\hslash }_{h}$ are [− 0.2, − 2.2], [− 0.3, − 2.3] and [− 0.6, − 2.4], respectively.

## Results and discussion

**Figure 3:**
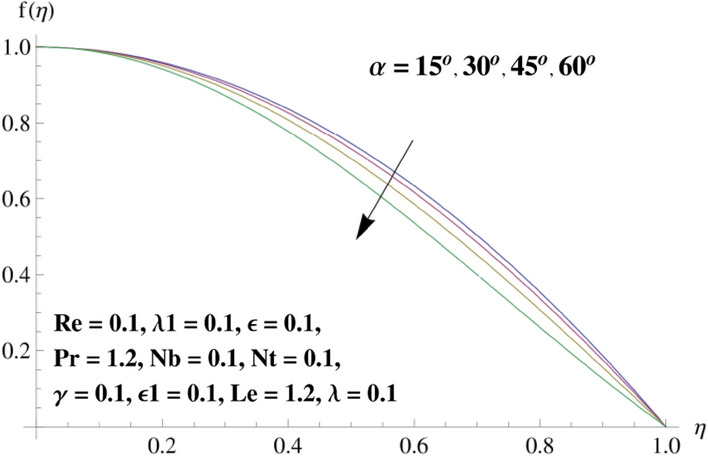
Behavior of $f\left( \eta \right)$ against $\alpha > 0$.

**Figure 4:**
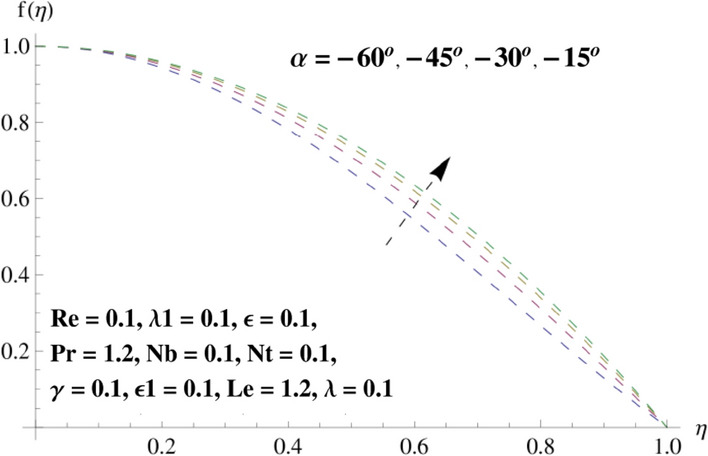
Behavior of $f\left( \eta \right)$ against $\alpha < 0$.

**Figure 5:**
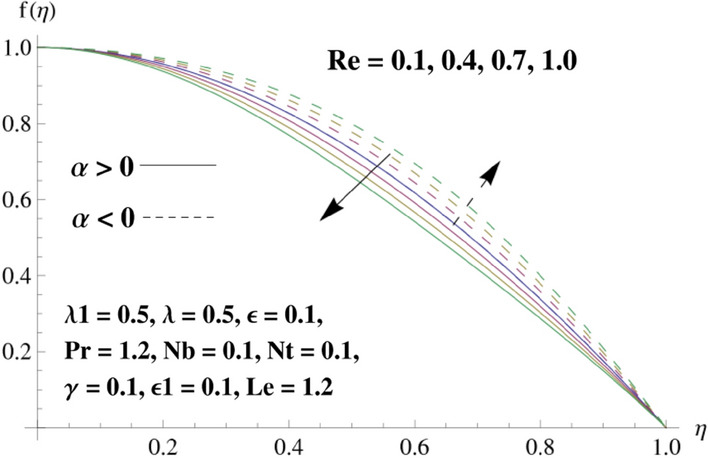
Behavior of $f\left( \eta \right)$ against $Re$.

**Figure 6:**
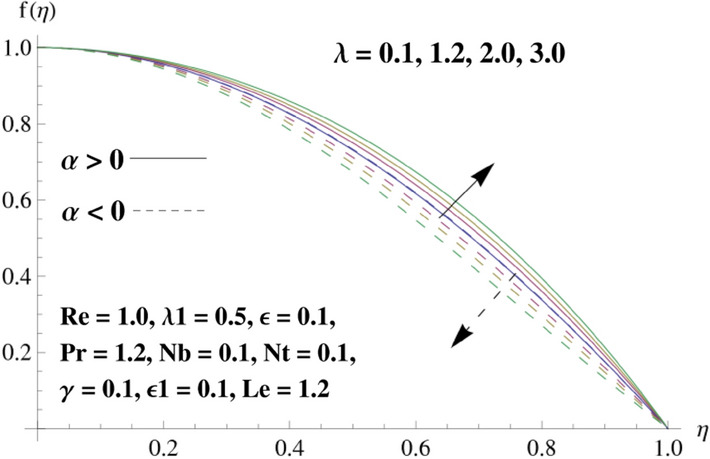
Behavior of $f\left( \eta \right)$ against $\lambda$.

**Figure 7:**
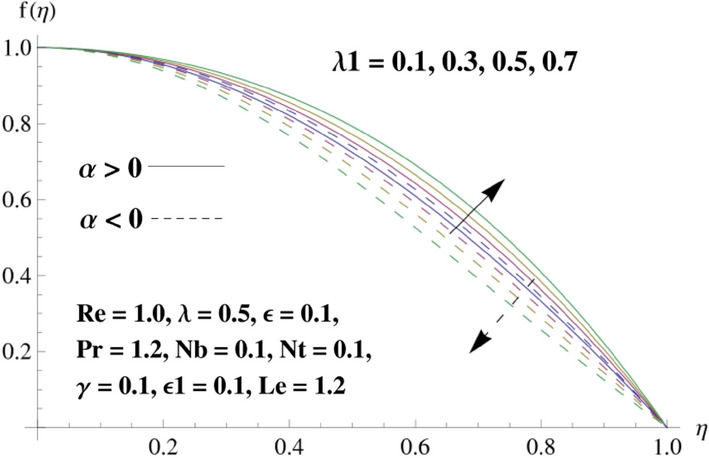
Behavior of $f\left( \eta \right)$ against $\lambda_{1}$.

**Figure 8:**
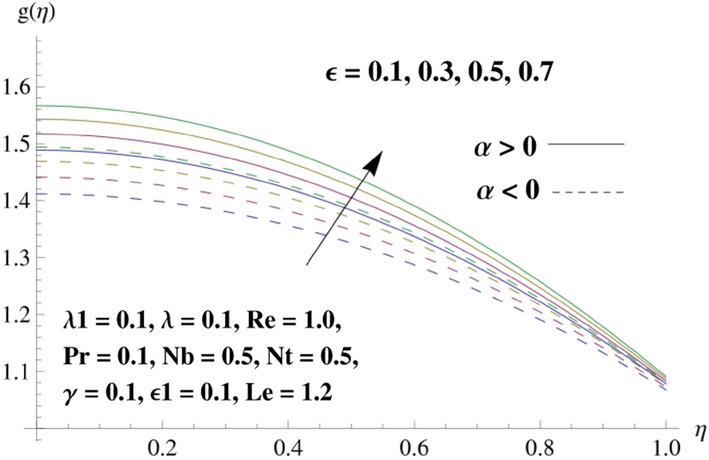
Behavior of $g\left( \eta \right)$ against $\in$.

**Figure 9:**
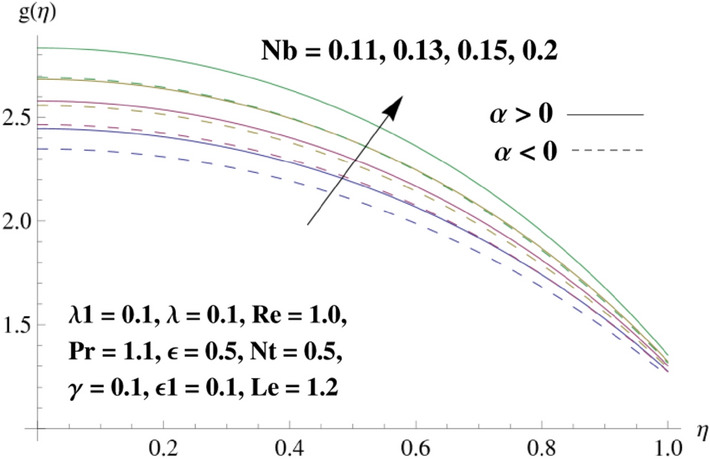
Behavior of $g\left( \eta \right)$ against $N_{b}$.

**Figure 10:**
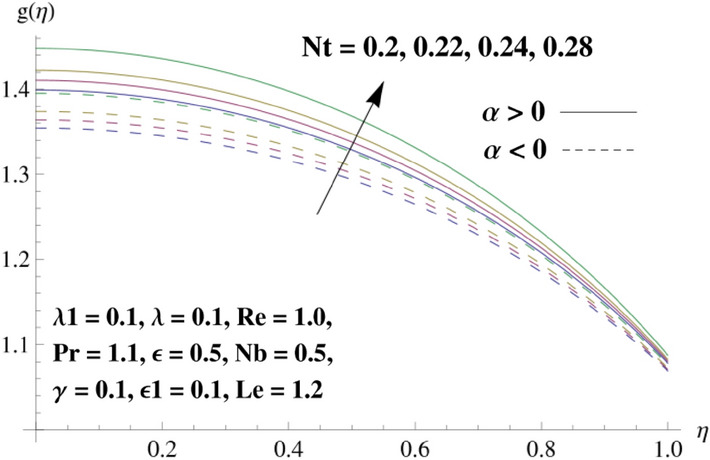
Behavior of $g\left( \eta \right)$ against $N_{t}$.

**Figure 11:**
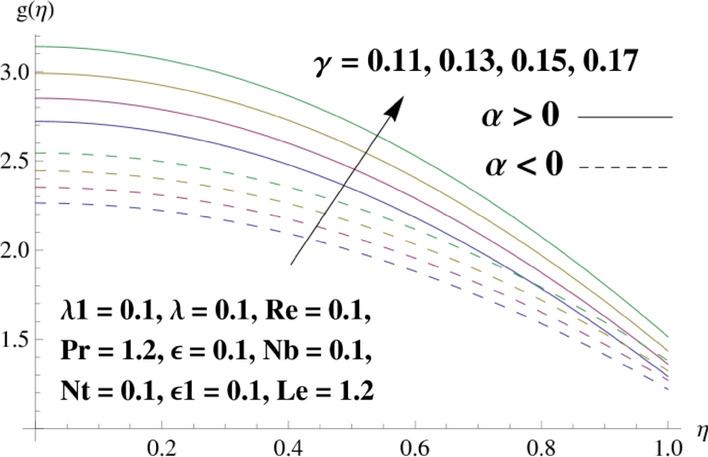
Behavior of $g\left( \eta \right)$ against $\gamma$.

**Figure 12:**
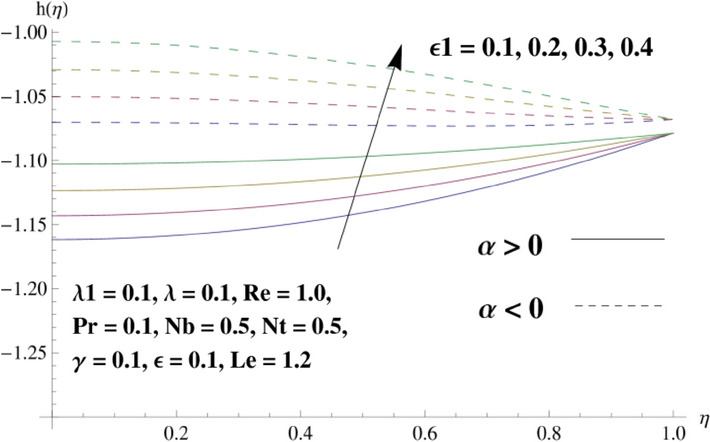
Behavior of $h\left( \eta \right)$ against $\in_{1}$.

**Figure 13:**
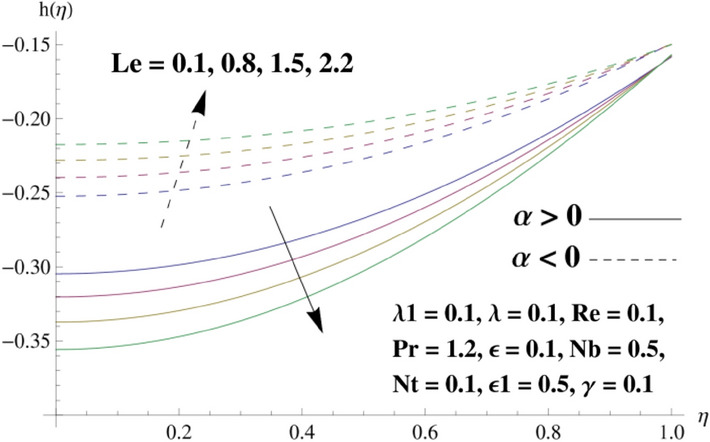
Behavior of $h\left( \eta \right)$ against $Le$.

**Figure 14:**
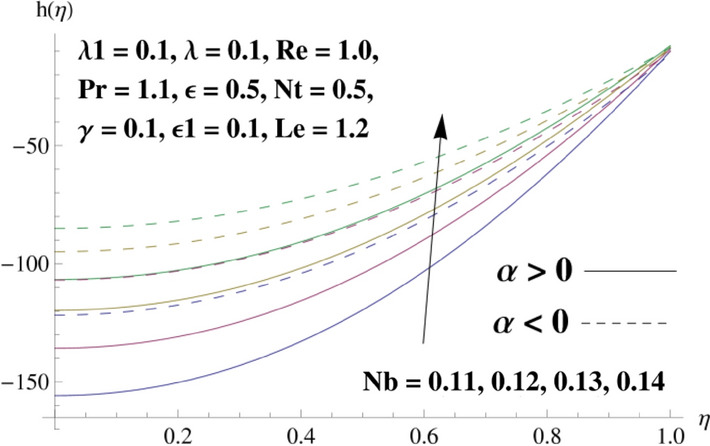
Behavior of $h\left( \eta \right)$ against $N_{b}$.

**Figure 15:**
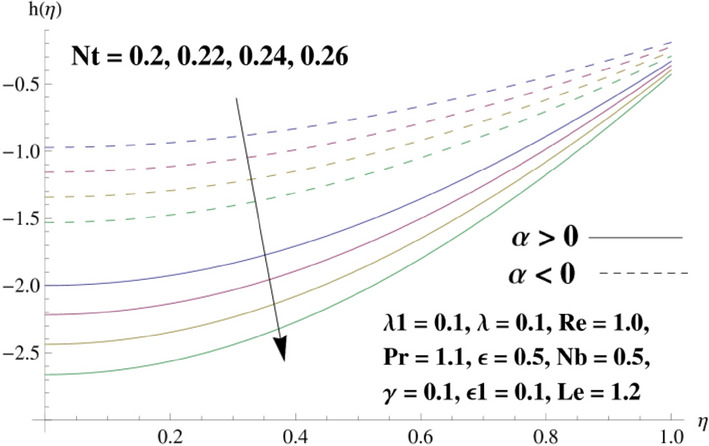
Behavior of $h\left( \eta \right)$ against $N_{t}$.

**Figure 16:**
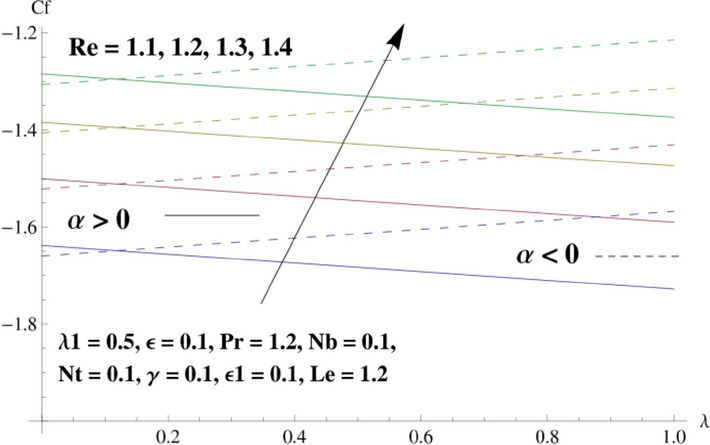
Behavior of $C_{f}$ against $Re$ and $\lambda$.

**Figure 17:**
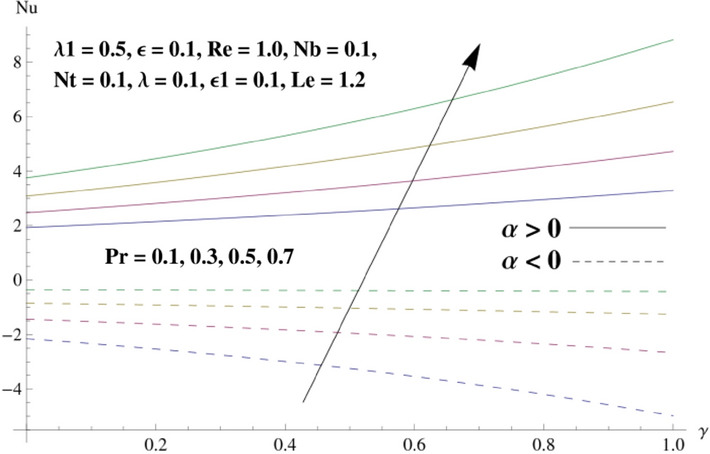
Behavior of $Nu$ against $Pr$ and $\gamma$.

**Figure 18:**
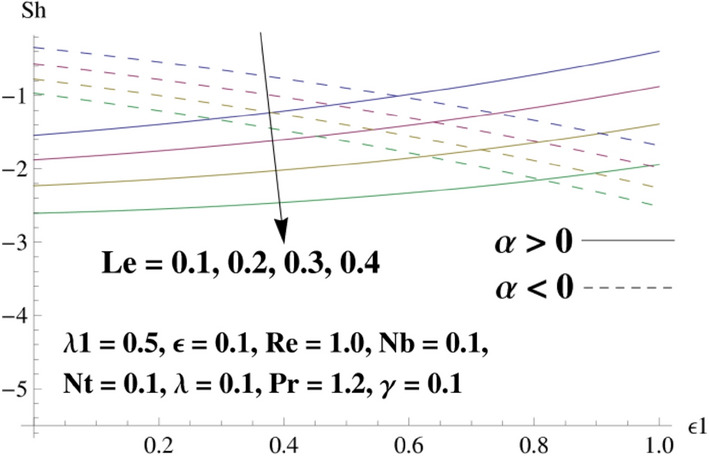
Behavior of $Sh$ against $Le$ and $\in_{1}$.

This portion is related with a graphical description of the emerged nanofluid characteristics on convergent/divergent flow. Here, in the plots, the solid lines represent divergent flow behavior for fixed inclination angle $\alpha =$ 30° while the dashed lines represent convergent flow behavior for fixed inclination angle $\alpha = -$ 30°. Impacts of the divergent channel angle $\left(\alpha >0\right)$ on velocity field are displayed in Fig. 3. It is reflected that velocity field is decremented by growing the angle. The channel angle has a dynamic role in the reduction of the fluid deformation phenomenon under the weak wall impact. From this phenomenon one can notice that away the non-parallel wall, weak wall impact resists the fluid movement and backward flow occurs, which consequences in an enhancement in frictional force. Hence, velocity field detracts. The physical features of the convergent channel angle on velocity field are predicted in Fig. 4. The magnitude of velocity is grown by enhancing the channel angle. These consequences reveal that a greater strength of the angle of inclination overcomes the effect of frictional forces and gives increasing response of velocity due to strong narrow wall effects. Analysis of the Reynolds number $(Re)$ on velocity field is depicted in Fig. 5. Here when Reynolds number is increased the velocity field grows for convergent flow while it decays for divergent flow. Physically, by increasing $Re$, drag forces under the divergent flow phenomenon amplify the fluid motion resistance away to the heated wall surface due to backward motion and as a consequence deceleration is obtained in the velocity field. On the other hand, fluid flow intensity due to larger $Re$ in case of convergent angle under the inertial forcre effects speed up the process of fluid deformation between the non-parallel walls and thus velocity significantly accelerates. The variation of velocity field versus the Darcy parameter $\lambda$ for both cases (converget and divergent) is plotted in Fig. 6. It is elucidated from Fig. 6 that for incrementing values of $\lambda$ the velocity field climbs in case of $\alpha >0$, and the for $\alpha <0$, velocity field diminishes. Here, it is noticed that dropping of velocity in convergent case is due to the strong resistive effects as strong $\lambda$ decreases the deformation rate of fluid and hence velocity is decremented. Furthermore, it is seen in divergent case that momentum thickness grows with the intensification of $\lambda$, which prompts that growing $\lambda$ corresponds to the strengthening of permeability effects and as a consequence, momentum thickness increments significantly. Moreover, strong permeability increases the rate of fluid deformation in the flow and consequently, velocity accelerates. Figure 7 depicts velocity variations for physical inertia parameter ${\lambda }_{1}$ in converging and diverging channel. It reveals that velocity field drops as inertia parameter increases in converging channel, while for divergent channel the flow description is converse. Physically, as the ${\lambda }_{1}$ rises, the surface drag becomes more significant, and resulting in reduced pore velocity which leads to a decrease in velocity in converging channel. Moreover, one can notice that escalating values of ${\lambda }_{1}$ result in faster fluid deformation as well as higher the flow rate. It is understandable that non-Darcy phenomenon aids the enhancement of permeability effects. Therefore, enhanced permeability effects raise the deformation rate which helps the flow velocity to amplify in a divergent channel. The impact of the thermal conductivity parameter $\in$ on fluid temperature is depicted in Fig. 8. With increasing $\in$, the temperature increments in the converging and diverging regions. Physically, these outcomes display that thermal conductivity parameter has a prominent role in growing the magnitude of temperature field. The figure reports that augmentation of ∈ , the thermal conductivity effects elevate with the boundary layer and therefore, increment in temperature field is witnessed. The Brownian diffusion parameter $({N}_{b})$ effects on temperature field are disclosed in Fig. 9. The magnitude of temperature is increased in both converging/diverging regions when ${N}_{b}$ is increased. In fact, the greater intensity of ${N}_{b}$ leads to more fluid's particles collision and consequently extra heat is being transferred from sheet to fluid. Therefore, temperature field rises. The thermophoretic parameter $({N}_{t})$ influences on the temperature field is reported in Fig. 10. The temperature is enhanced in converging/diverging regions by growing ${N}_{t}$. It is seen that molecular collisions can be helpful to strengthen the thermophoretic diffusion characteristics in order to intensify the heat transport effects. Incrementing the values of ${N}_{t}$, boosts up the phenomenon of fluid's particles pull from plate towards fluid and consequently acceleration in temperature field is witnessed. Figure 11 displays the temperature variations for thermal slip parameter $\gamma$. It is noticed that growing $\gamma$ upsurges the temperature field in diverging/converging channels. Physically, when $\gamma$ is enhanced, a significant amount of heat is shifted from heated surface towards adjacent fluid, and therefore, temperature field upsurges. The impact of the mass diffusivity parameter ${\in }_{1}$ on concentration field is depicted in Fig. 12. With increasing ${\in }_{1}$, the concentration increments in the converging and diverging regions. Physically, these outcomes display that mass diffusivity parameter has a prominent role in growing the magnitude of concentration field. The figure reports that augmentation of ${\in }_{1}$, the mass diffusivity effects elevate with the boundary layer and therefore, increment in concentration field is witnessed. Figure 13 illustrates the deviation in concentration field when Lewis number $Le$ varies. Concentration field decays in divergent channel due to increment in $Le$. Physically uplifting Lewis number corresponds to the diminishing of the mass diffusivity and as a consequence, concentration field weakens significantly. Figure 14 depicts the impact of Brownian diffusion parameter $({N}_{b})$ on concentration field. It is seen that concentration field is enhanced with the incrementing of ${N}_{b}$ in both channels. In fact, large ${N}_{b}$ produces more molecular diffusion within the channels, consequently the concentration field upsurges. Figure 15 reports concentration variations against thermophoretic parameter $({N}_{t})$. Improved ${N}_{t}$ values cause concentration field to decline in both converging/diverging channels. Physically, incrementing thermophoretic parameter ${N}_{t}$ is responsible for weak diffusive force which ultimately diminishes the phenomenon of mass diffusion and thus, concentration field becomes lessen. Figure 16 displays the variation of $Cf$ (skin friction) against the Darcy $(\lambda )$ and Reynolds $(Re)$ numbers. It is elucidated that increasing $\lambda$ enhance the magnitude of ${C}_{f}$ in both channels but opposite trend is witnessed against $Re$. Figure 17 illustrates Nusselt number $Nu$ variation with the impact of Prandtl number $Pr$ and thermal slip parameter $\gamma$. Intensification in $Pr$ enhances the Nusselt number whereas decrement is seen in Nusselt number for larger $\gamma$. Figure 18 displays the variation of $Sh$ (Sherwood number) against the Lewis number $(Le)$ and mass diffusivity parameter $({\in }_{1})$. It is elucidated that increasing $Le$ and ${\in }_{1}$ reduce the $Sh$ in both channels. The computations of co-efficient of skin friction against various pertinent parameters are depicted in Table 1. The table reveals that greater ${\text{Re}}$ diminishes the skin friction coefficient while increasing $\lambda$ and $\lambda_{1}$ intensifies the skin friction coefficient in both channels. Furthermore, skin friction coefficient shows prominent behavior in convergent channel as compared to divergent channel. In Table 2, the present results have been compared for skin friction $\left( {Cf} \right)$ with those disclosed in the past study^39,40^. An excellent agreement is noticed between the studies.

**Table 1:** Values of Coefficient of Skin friction ($Cf$) for diverse values of ${\text{Re}} ,\lambda$ and $\lambda_{1}$ when $\in \; = \; \in_{1} \; = \;Nb\; = \;Nt\; = \;\gamma \; = \;0.1,\;Le\; = \;\Pr \; = \;1.2$.

${\text{Re}}$	$\lambda$	$\lambda_{1}$	$Cf$ (*α *= 18°)	$Cf$ (*α *= − 18°)
1.1	0.1	0.9	− 1.7569	− 1.7590
1.2			− 1.6104	− 1.6125
1.3			− 1.4865	− 1.4886
1.4			− 1.3802	− 1.3823
0.4	0.1	0.9	− 4.8334	− 4.8355
	0.3		− 4.8440	− 4.8248
	0.5		− 4.8546	− 4.8142
	0.7		− 4.8652	− 4.8036
0.4	0.5	0.1	− 4.8039	− 4.8650
		0.4	− 4.8229	− 4.8460
		0.7	− 4.8419	− 4.8269
		0.9	− 4.8546	− 4.8142

**Table 2:** $f^{\prime}\left( 1 \right)$ via $\alpha$ and $\beta$ when ${\text{Re}} = 50$ and $\lambda = \lambda_{1} = 0$.

*α *= 5°				*α *= − 5°			
$\beta$	Turkyilmazoglu^39^	Mohyud–Din et al.^40^	Present	$\beta$	Turkyilmazoglu^39^	Mohyud–Din et al.^40^	Present
− 1	− 3.508103	− 3.508103	− 3.508103	− 1	− 3.508103	− 3.508103	− 3.508103
− 0.5	− 2.173044	− 2.173044	− 2.173044	− 0.5	− 2.173044	− 2.173044	− 2.173044
0	− 1.109326	− 1.109326	− 1.109326	0	− 1.109326	− 1.109326	− 1.109326
0.5	− 0.361846	− 0.361846	− 0.361846	0.5	− 0.361846	− 0.361846	− 0.361846
1	0.000000	0.000000	0.000000	1	0.000000	0.000000	0.000000

## Conclusions

The current research is dedicated to exploring the thermal jump and variable fluid characteristics in a steady two-dimensional Jeffrey–Hamel nanofluid flow under non-Darcy permeable medium. The zero mass flux condition is considered at boundary wall. The present investigation indicates that increasing Darcy and inertia parameters raise the velocity field in divergent channel, while converse trend is noticed in convergent channel. By the rise in thermal conductivity and mass diffusivity parameters, the temperature and concentration fields are increased respectively. Fluid temperature is weaker for thermal slip parameter. It is anticipated that the current investigation arises in much realistic implications, for instance, environmental, petroleum, and groundwater hydrology, molten polymers extrusion via converging dies, cold drawing operation related to polymer industry, medical treatment, processing plants, automobile etc. Future researches will prompt incentive more complex fluid such as rate type fluid, power law nano-liquids, Furthermore, three-dimensional case can also be tackled.

## Data Availability

All data generated or analyzed during this study are included in this published article.

## List of symbols

$u$: Velocity component, ms^−1^
$U_{c}$: Centerline velocity, ms^−1^
$\mu_{f}$: Nanofluid absolute viscosity, kgm^−1^ s^−1^
$\rho_{f}$: Density of nanofluid, kgm^−3^
$$p$$: Pressure, kgm^−1^ s^−2^
$k^{*}$: Permeability of porous medium, m^2^
$k_{\infty }$: Thermal conductivity, Wm^−1^ K^−1^
$$D_{T}$$: Thermophoretic diffusion, m^2^s^−1^
$\gamma$: Thermal slip parameter
$$T$$: Temperature, K
$$T_{w}$$: Wall temperature, K
$c_{p}$: Heat capacity, Jkg^−1^ K^−1^
$$\alpha_{f}$$: Thermal diffusivity, m^2^s^−1^
$$T_{\infty }$$: Ambient temperature, K
$$D_{B}$$: Mass diffusivity, m^2^s^−1^
$D_{\infty }$: Mass diffusivity surrounding, m^2^s^−1^
$$Le$$: Lewis number

## Dimensionless parameters

$$\lambda$$: Darcy number
$$\Pr$$: Prandtl number
$\in$: Thermal conductivity parameter
$\in_{1}$: Mass diffusivity parameter
$N_{b}$: Brownian motion parameter
$C_{f}$: Skin friction coefficient
$$\eta$$: Similarity variable
$$\lambda_{1}$$: Inertia parameter
$$C$$: Concentration
$${\text{Re}}$$: Reynold number
$$Nu$$: Nusselt number
$$N_{t}$$: Thermophoresis parameter
$$Sh$$: Sherwood number
